# A systems approach to creative flourishing: conceptual foundations and implications for development

**DOI:** 10.3389/fpsyg.2025.1518993

**Published:** 2025-09-12

**Authors:** Cordele Glass

**Affiliations:** School of Creative Human Development, Catalyst Institute for Creative Arts and Technology, Berlin, Germany

**Keywords:** creativity, creative development, creative flourishing, flow, experiential education, Creative Agency, Creative Self-Efficacy

## Abstract

This article introduces the term “Creative Flourishing” defined as the experiential confluence of Creative Agency (one's drive to create), Creative Self-Efficacy (belief in one's creative ability), and Flow Proneness (the tendency to experience flow during creative activities) within an appropriate and responsive environment. When an individual experiences each of these aspects together they can assert that they are driven to create, they feel they have the ability to be creative, and they enjoy creating. Thus, Creative Flourishing is the result of the harmonious alignment of one's creative desires, self-perception of skills, and experiences within their context. This paper synthesizes existing literature to define Creative Flourishing, reviews intervention strategies aimed at cultivating awareness of its components, and discusses potential measures for assessing growth in these areas.

## Introduction

The phenomenological experience of creating something new, whether through problem solving, or self expression, is fundamental to our health and wellbeing (Runco, 2021). Improving awareness of developmental systems and creative processes can play an important role in the development of these creative experiences (Acar et al., 2021). This process can be energized through the help of interventions which provide direct participatory experiences and opportunities to reflect on those experiences. This reflection will help participants draw new conclusions and personal insights about themselves and the world around them (Kolb, 2014). Thus, if a program or intervention seeks to improve the creativity of its participants, then improving awareness of the components involved could lead to significant developmental changes.

This paper outlines approaches to cultivating creative awareness, evaluates contemporary strategies for fostering creative flourishing, and examines assessment methods for educational interventions in creativity. Researchers have attempted to conceptualize what it means to be “more creative” for decades. In the behavioral era of psychology a large emphasis was of course placed on creative behaviors and material outcomes of those behaviors (Feist and Runco, 1993; Hennessey, 2015; Ryhammar and Brolin, 1999; Sloane et al., 1980). This paper adopts a phenomenological perspective, focusing instead on the subjective experience of creativity. From this phenomenological perspective, creative flourishing refers to the experience of positive engagement with the creative process and its contributing systems.

Through an examination of contemporary literature the author will offer a systems model of creative flourishing that will help to synthesize and integrate the many factors involved. This is especially helpful for educators, coaches, therapists, and change agents who are looking for informed guidance on how to foster wellbeing for creative individuals and communities. Systems models provide a unique lens through which practitioners can make informed decisions that take many factors into account without being so complex as to inhibit decisive action. Although the Systems Model of Creative Flourishing draws heavily from contemporary evidence-based research it has not yet been validated with studies of its own. This is a possible area of future research which could serve to strengthen the model even further.

## Creativity through the lens of systems

In our modern scientific landscape systems models have come to dominate a wide variety of theories and models across fields such as engineering, psychology, political science, architecture, and more (Jackson and Moraes, 2023). Major problems of our time like energy, food security, the environment, and food security are largely understood as systemic problems (Capra and Luisi, 2014; Midgley and Lindhult, 2021; O'Day and Smith, 2016). Thinking in systems is also integral to the fields of creativity and developmental science, although this has not always been the case. In the field of Positive Psychology, under which creativity scholarship tends to fall, early researchers' work began with criticism for being too narrowly focused on positive aspects of human experience such as happiness, gratitude, and positive emotions (Wong and Roy, 2017).

This “First Wave” of Positive Psychology was supplanted by a “Second Wave” of Positive Psychology in which the importance of negative emotions and difficult experiences were given their due attention in the conversation around human flourishing. Research on concepts like resilience and post-traumatic growth began to show that some difficult experiences can actually result in a deeper appreciation for life, more meaningful personal relationships, and an increased sense of personal strength (Linley and Joseph, 2005; Seery, 2011; Tedeschi and Calhoun, 2004). In addition to difficult experiences, the utility of negative emotions also becomes more common in research and practice (Lomas and Ivtzan, 2016). But still, even with this broader integration, the field was considered too narrowly focused for the lofty goal of articulating the elements needed for a life well lived.

This progression has culminated in the contemporary “Third Wave” of Positive Psychology (Lomas et al., 2021; Van Zyl and Salanova, 2022) in which a systems theory approach reigns supreme. In this era the reciprocal relationships between diverse elements of life and their integration become the focus of theory, research, and application. This systems-oriented evolution is consistent with (Wong 2011)'s Positive Psychology 2.0 and the broader complexity-informed approaches now seen in education, clinical work, and developmental psychology (Lomas et al., 2021; Seligman and Csikszentmihalyi, 2000). Modern theoretical approaches like Systems Informed Positive Education which emphasizes holistic educational practices (Kern and Taylor, 2021), Systems Informed Positive Psychology (SIPP), which promotes psychological interventions aimed at many diverse factors of life (Kern et al., 2020), and Developmental Systems Theory (Molenaar et al., 2013) which highlights the vast landscape of elements implicated in human development, all bring an emphasis on aligning complex systems to operate in harmonious ways.

As such, any modern attempt to explain creative flourishing will take a systems approach to understanding not only the various parts involved in the system, but also their relationships to each other and the emergent properties that manifest when the system is in harmony or dysregulation. The following section will review the current landscape of creativity models that make use of a systems theory approach. This will help to inform our broader systemic view of creativity when we explore how to apply this approach to the phenomenological experience of creative flourishing.

## A systems model of creativity

Dr. Mihalyi Csikszentmihalyi, one of the most eminent creativity scholars in the field, has developed a vast and in-depth view of creativity over his lifetime of research. He is most well known for his work on the Psychology of optimal experience, otherwise known as flow states, in which people become deeply absorbed in an activity (Nakamura and Csikszentmihalyi, 2002), but his research also reaches deep into the intersection of creativity and complex systems. In what he calls The Systems Model of Creativity (Csikszentmihalyi, 2014) he posits that we cannot study creativity by isolating individuals and their works from the social and historical milieu in which their actions are carried out. This theoretical approach goes beyond a narrow focus of merely the individuals involved in creativity, or even the products created by creative individuals. Instead, he proposed three broad categories which, when integrated systemically, fully encompass the broad topic of creativity.

“The Domain” is a cultural amalgamation of ideas, forms, techniques, and artifacts that are transmitted throughout generations. Tools, materials, approaches, and techniques that shape the way people engage with the creative process are all crucial for understanding creativity more broadly. These are the things people are taught by instructors, the art that is preserved in galleries, the music perpetuated in conservatories, and the recipes passed down from ancestors. Although the techniques and artifacts found in any given domain are essential for creativity, access to these domains is historically uneven. Prejudice and discrimination shaped by education, socioeconomic status, cultural differences, and the distribution of resources and cultural capital have been known to marginalize or even explicitly exclude many people from creative domains (Banks, 2007; Eisner, 2002; Johnson and Bourdieu, 1993; Magni et al., 2024). The elements within any particular domain from music to cooking to painting and beyond all play a critical role in shaping how any individual would connect with the creative process.

“The field” is a set of institutions that selects from the variations produced by individuals to determine which are worth preservation (Csikszentmihalyi, 2014). This includes individual gatekeepers, tastemakers, and decision makers, but also broader institutions such as galleries, record labels, publishers, or any organization which participates in the selection of ideas to be included within a domain. To consider creativity without considering the social environment around the creative process is to miss a massive portion of context which shapes the creative landscape (Banks, 2007). Unfortunately, the decision-making power wielded by members of a given field can often be abused. This is where systemic injustices and biases can have a negative impact on individuals who are attempting to contribute to their respective domains.

Finally, we have “the individual,” who brings about change within domains. The individual, their personality, personal tastes, motivations, and interests all interact with the creative domain in which they participate. This inevitably leads to variations of the domain as individuals create, design, compose, and make new things. These new variations are then considered by the field either too derivative or too avant garde, in which they are ignored or discarded. Alternatively, the new variations might be considered brilliant, moving, and deeply engaging, in which case they are perpetuated and upheld through inclusion in the domain.

This model is incredibly useful at understanding the vast scale of the creative process, but it does very little to highlight the personal experience an individual may have while navigating this complex system. In order to more fully grasp the experience of creative flourishing a new model, like the one suggested in this paper, may be helpful in examining the more immediate experiences and psychological factors which contribute to a deep sense of creative flourishing.

## The creative-being model

The Creative-Being Model by (Beresford et al. 2024) provides a framework for understanding how mental health and relational dynamics contribute to creative flourishing. Their model examines the interactive relationships between Psychological Wellbeing, PERMA, negative emotions, reflection, and positive relationships. The PERMA model, developed by (Seligman 2011), outlines five core pillars of wellbeing: Positive Emotion, Engagement, Relationships, Meaning, and Accomplishment.

The Creative-Being model was designed by educators with application in mind throughout the design of the model. This is helpful for understanding the impact of the teacher-learner dynamic in the process of creative wellbeing development. This model draws heavily on Dr. Amabile's “Componential Model of Creativity” (Amabile and Pillemer, 2012; Amabile and Mueller, 2024) which includes domain-relevant skills such as talent, and expertise. It also includes creativity-relevant processes such as personality traits like persistence and curiosity, as well as intrinsic motivation, affect, and social context.

(Beresford et al. 2024) apply the model within creative education settings, finding improved creative output and wellbeing in their students. To date, replication of empirical findings from the model in educational settings remains limited, though related pedagogical approaches show promise (Darewych, 2019; Reeve and Cheon, 2024; Reeve et al., 2021). The model is applied within classroom contexts with an emphasis on positive connections between students and teachers. This helps to promote emotional vulnerability within an emotionally safe environment. This positive connection allows learners to attempt new tasks, share new ideas, and learn from their mistakes. These experiences are paired with self-reflection and a prioritization of their own wellbeing to enable learners to creatively thrive.

A model which emphasizes learning experiences and wellbeing relationships is incredibly valuable in the effort to enhance creative flourishing, however, like Csikszentmihalyi's systems model of creativity it does not highlight the personal experiences one has while effectively engaging in creativity systems. It comes close with its connections to wellbeing and PERMA, but there is still room for an even more granular and personal approach to the experience of flourishing within a creative practice.

## A Subjective Creative Wellbeing Suprasystem

Shields' (2017) model of a Subjective Creative Wellbeing Suprasystem offers a distinct perspective by emphasizing the subjective experience of creative wellbeing across interconnected self and environmental systems. In his model he identifies a self-system, an environmental system, and an intermediary system—each of which contains their own various subsystems.

The Self System has four subsystems. These include the Physiological Dimension which accounts for embodiment and somatic functions, the Affective Dimension which represents deliberate emotional and spontaneous emotional psychological creative processes, the Social Dimension which accounts for the dynamic process of shaping the self in the socio-cultural environment, and finally, the Cognitive Dimension which accounts for processes of knowledge acquisition, such as perception, reasoning, intuition, and problem-solving.

The Environmental system has three subsystems. These include the Physical Environment system which accounts for human-made (social space, cultural representations) and natural environments in which humans live, learn, and work, the Temporal Environment system which accounts for the different ways in which individuals and groups experience the occurrence of events in time, and finally, the Social Environment subsystem which accounts for social forces that shape beliefs, values, goals and behaviors.

Finally, there are three Intermediary Systems which act upon each of the other subsystems. These include the Motivation System which represents all conscious and subconscious behavior as being motivated, the Self-Regulation System which encompasses stress, coping methods, and social support, thoughts, and behavior, and finally, the Contingent Assemblages System which represents maps of power relations, which imperceptibly shape possibilities of behavior, thought, and language.

Of the models discussed thus far this comes the closest to touching on the personal experience of creative flourishing with its examination of affect, perception, and cognition. It addresses the psychological factors that contribute to the experience of creative flourishing but it stops short at addressing the experiences themselves. A systems model that takes this next step could address the phenomenological experience of these systems when they are functioning optimally. These models offer partial insights into the systemic nature of creativity, but none fully address the lived experience of creative flourishing as this model aims to do.

### A systems model of creative flourishing—An experiential perspective

**Figure 1:**
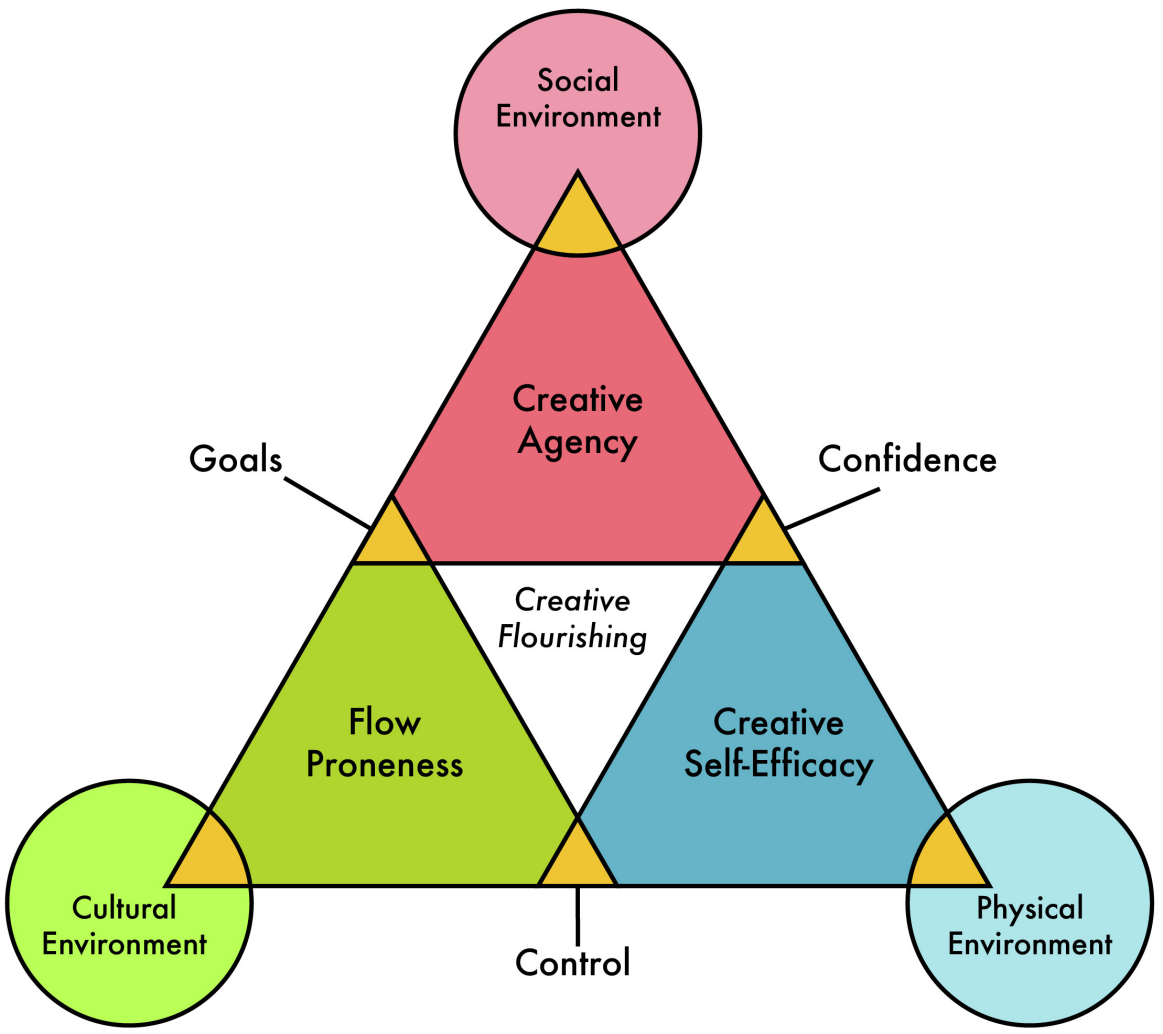
A systems model of creative flourishing.

Building upon the previously described systems models of creativity, the rest of this paper will explore the details related to the Systems Model of Creative Flourishing. This model seen in Figure 1 uses a systems perspective to examine the relevant phenomenological aspects of creative flourishing. In other words, what does it *feel* like to creatively flourish? This is in contrast to other conceptualizations which focus on more material aspects of creativity such as social prestige or highly praised creative products.

This new model is helpful for several reasons. The first is that it gives teachers, coaches, therapists, and other change agents a framework for understanding the subjective experience of those they are working to support in a creative context. This will allow for deeper empathy and social connection which has been shown to be one of the most important factors related to successful intervention outcomes (Ardito and Rabellino, 2011; Baier et al., 2020).

This model is also helpful in determining how to measure and evaluate interventions aimed at improving people's experience of creating. Operationalizing positive creative experiences and identifying specific methods of measurement is a crucial process for any type of educational or therapeutic program interested in maintaining an evidence-based and research-informed approach. The Systems Model of Creative Flourishing identifies several areas of measurement that already have validated scales and measurement tools which can be used and repurposed for more specific contexts. The following sections will explore each aspect in more detail.

This model seen in Figure 1 is composed of three interacting elements: Creative Agency (one's drive to create), Creative Self-Efficacy (belief in one's creative ability), and Flow Proneness (the tendency to experience flow during creative activities). Each element is distinct and yet as a complex system they are also deeply integrated and reciprocal. Rather than rely on an abundance of arrows as many systems models tend to do, this model opts for a cleaner design via a modified Venn diagram which highlights systemic interactions through overlapping shapes. Creative Flourishing occurs when each of these elements are working in harmony together with positive subjective experiences. If any one of these elements are compromised and leading to difficult subjective experiences then we may consider the individual low in creative flourishing.

While each of the components—Creative Agency, Creative Self-Efficacy, and Flow Proneness—has been studied independently, they have not yet been combined into a systems-based experiential framework focused explicitly on the subjective feeling of flourishing during creative activity. The novelty of the present model lies in this synthesis, and in its emphasis on dynamic, moment-to-moment experience rather than output, skill, or achievement alone.

As we know from systems informed theories, there is more to creativity and wellbeing than simply the individual. As such, each element has a direct and reciprocal interaction with the environment around the individual. This means the environment shapes each element of the individual and in turn the individual shapes their environment through these elements.

While the interrelationships between these dimensions may appear intuitive, the model's contribution lies in framing them as part of a recursive, self-reinforcing system that operates across different ecological levels—internal (beliefs, emotion, attention), relational (feedback, expectations), and environmental (physical and cultural). The model does not claim to reinvent the components but to offer a phenomenological systems lens that can inform applied research and intervention.

## Creative agency

Creative Agency refers to the psychological properties involved in taking autonomous ownership and deliberate control over one's creative endeavors. The bulk of the research done on psychological agency has come from Dr. Albert Bandura who describes someone with agency as one who is able to intentionally influence one's functioning and life circumstances. Agency allows one to be generative, creative, proactive, and reflective, not just reactive (Bandura, 2023).

More recently, (Sternberg 2018) has built upon this idea of creative agency by highlighting the creative attitudes and decisions involved in creativity. In his Triangular Theory of Creativity creative choices are specifically defined as defying some combination of crowds, the self, and the cultural zeitgeist. This kind of deliberate defiance can be seen as another example of the creative process requiring some form of agency.

Creative agency is simply psychological agency in the context of creative practice and experiences. Creative behavior results from a person's intentional actions, which are influenced by that person's belief system. Consequently, the movement from creative potential to creative behavior represents an agentic action (Karwowski and Beghetto, 2019). Deciding to behave creatively, from an agentic perspective, is considered to be a necessary precondition for reliable creative performance. This is not to say that a person who decides to behave creatively necessarily has an explicit awareness of this decision (e.g., “*I am deciding to act creatively now*”). Rather, an agentic perspective simply asserts that the person has decided to think and act in a new or different way.

In his work on Social Cognitive Theory (Bandura, 2023) identified three properties of agency. The first is known as Forethought. This involves future-oriented intentions, plans, strategies, and goals. Someone high in Creative Agency will have a clear vision for the future and they will understand the roles they desire for themselves in that imagined future.

The second element involved in agency is Self-Reactiveness. This refers to the self-regulation involved in setting personal standards of behavior. Self Reactiveness also involves reacting to one's own behavior as it relates to those personal standards. Often implicated in moral judgments and goal-based motivations, self-reactiveness serves to align an individual's behavior with their own forethought.

Finally, agency involves Self-Reflection which is the metacognitive capability to reflect on oneself and on the adequacy of one's capabilities, thoughts, and actions. Self-reflection is a higher, more meta level of thought than self-reactiveness in which the future, the past, and one's place in it is considered, especially as it relates to meaning, values, morality, and one's personal goals. This process of self-reflection is what allows the system to become self-governing, make course corrections, and consistently improve over time.

### How creative agency relates to the environment

Creative Agency is deeply connected to our self-concept and sense of self, which is primarily a social process (Bandura, 1997). Agentic development moves beyond merely recognizing oneself as an agent in the world and extends to recognizing oneself as a distinct person in relation to other distinct people. This process is integral to developing a self-identity that is both unique yet embedded within a broader social context. This personal identity is in direct relationship with one's social identity. Our sense of self is constructed through the way we are treated by significant others. The people around an individual perceive, socially label, and treat one as the same person over the course of life despite physical changes. These social interactions work to construct individuals with varying levels of agency and autonomy.

But the relationship between an individual and a social environment is not a one-way interaction. Although social relationships and behaviors have a direct impact on an individual's sense of self, that very same individual, through their own sense of agency, can directly influence the social environment as well. Techniques such as adaptive distancing from specific individuals (White et al., 2015), setting clear boundaries, and even intentionally choosing specific social contexts via moving schools, jobs, or cities are all ways to change one's social environment. Acting on a strong sense of agency has even been shown to help tailor experiences of social media and other online social interactions (Ryan and Linehan, 2022).

### Improving awareness of creative agency

Becoming more aware of the mechanisms involved in developing creative agency, as well as the potential benefits of its development, could allow individuals to invest more time and energy into their own creative practice in ways that improve their overall wellbeing and creative flourishing.

Programs, curricula, or interventions that highlight causal relationships between individuals and their creative environments can help to develop a deeper sense of agency (Bandura, 2023). This process begins in infancy with simple actions such as seeing a ball knock a bottle off of a table. As we grow older these causal relationships can get much more complex, especially as they relate to topics like culture, politics, and economics. Recognizing that an individual's creative process can have a direct causal relationship to systems as vast as these is integral to developing a broader sense of creative agency.

Beyond the recognition of causal relationships, agency development involves the recognition that causation takes place through actions. This may be a recognition that the actions of others lead to causal outcomes, but it also involves recognizing that one's own actions lead to causal outcomes. In a creative context for example, beyond the recognition of paint and canvas causing patterns to appear, one may recognize that the action of painting is what led to the outcome of patterns on a canvas. On a broader scale one might recognize that the action of creating a piece of art with a meaningful message may have a direct causal relationship to political change.

Finally, a sense of agency becomes more fully formed when one recognizes themselves as the initiator of these causal actions. Recognizing oneself as a unique individual who can take autonomous action to create specific causal outcomes is at the core of agentic experiences. Programs aimed at improving awareness around creative agency could allow ample opportunities for participants to set personal intentions. This will allow participants to practice enacting their own creative agency in real world contexts.

### How creative agency is measured

Considering the subjective and phenomenological nature of creative agency, a qualitative design could be a good fit for measurement. This approach is concerned with meaning, putting experience at the center of the research, and allowing the participants to share their experiences in a more natural way (Willig, 2013). Qualitative research seeks to understand and interpret patterns, while accommodating conflict in the data and embracing the complexity of human experience (Braun and Clarke, 2013). It can provide a comprehensive understanding of individual experience through careful examination of goals, motivations, and expectations of behaviors, while remaining open to multiple interpretations and meanings. For example, (Ryan and Linehan 2022) used the framework of personal agency to conduct semi-structured interviews using a critical realist qualitative research design.

As for quantitative measurements of creative agency, The Experience of Creativity Questionnaire (Nelson and Rawlings, 2009) has been used to measure very similar phenomena. This questionnaire gathers information that is more phenomenologically rooted and was developed from previous qualitative research findings that were collected on an artist sample. Specifically, it examines the ways creativity is experienced and the existential meaning derived from the creative experience.

The Experience of Creativity Questionnaire has eight scales, however, the three existential scales stand out as most related to creative agency. The first is the Centrality of the Creative Process scale, which is directed toward self-discovery; it explores the addictive quality of engaging in the creative process as well as a strong desire to connect to some form of meaning. The Transformation scale investigates the sense of deep engagement with the self and the world. It explores the heightened awareness of confidence and the sense of healing that is derived from the creative process. The Beyond the Personal Scale examines the creative desire to expand beyond the personal realm of the individual or group; it is the desire of the creators to reach a broader audience. Each of these touch on topics of identity, sense of self, and personal agency.

Another potential way to measure creative agency lies with the Creativity Motivation Scale (CMS) (Zhang et al., 2018). This scale is made up of three subscales of three creativity-related behaviors (doing, learning, and accomplishing new things). It also includes three forces (high-quality experience, instrumental purpose, and value). For example, a sample item with the combination of doing new things for high-quality experience force is: “*I experience pleasure when I discover new things I've never seen before*.” This scale helps to identify the personal motivational factors that drive an individual's agentic goals and behavior.

## Creative self-efficacy

Creative Self-Efficacy is defined as “*the belief one has the ability to produce creative outcomes*” (Tierney and Farmer, 2002). The concept reflects a self-judgment of one's own creative capabilities or potential. This perception of one's skills has a direct impact on an individual's activity choice, effort exertion, and ultimately, the attainment of innovative outcomes.

Creative Self-efficacy is often conflated with another similar and related concept known as Creative Self-Concept which refers to the belief in one's ability to think or act creatively in and across particular performance domains (Karwowski and Beghetto, 2019; Li et al., 2022). Creative self-concept and creative self-efficacy are both creative confidence beliefs, but the former is more general, stable, and holistic, while the latter is relatively particular, malleable, and future-oriented (Beghetto and Karwowski, 2017). Creative Metacognition (CMC) is also deeply tied to Creative Self Efficacy. CMC helps to regulate behavior during creative activities by placing awareness on one's own thoughts about the process in order to make appraisals about their skills and strategies (Karwowski and Kaufman, 2017).

Creative Self-Efficacy is a crucial ingredient to Creative Flourishing because even with all of the most motivating and appropriate goals in the world, if one feels like they are incapable of achieving them then they are unlikely to even attempt them. This self-fulfilling prophecy can lead to a downward spiral in which one believes they cannot accomplish a creative goal or vision, so they make no attempt to do so, which then leaves them with absolutely no evidence that they could achieve the goal (Romney et al., 2024). This lack of evidence can then be used to further reinforce the idea that they cannot achieve a particular goal or vision which may lead to a downward spiral of diminishing self-beliefs.

### How creative self-efficacy relates to the physical environment

Creative self-efficacy beliefs are not statements of intentions of what one will do, they are not abstract conceptions of one's “skills” nor are they feelings of self-worth. Self-efficacy beliefs are judgments of what one can do in a current or prospective situation (Bandura, 1986). What one can or cannot do will always be constrained by the physical environment around them. In a creative context, even if one has all of the belief, intention, and skill in the world for painting, if they have absolutely no access to any form of painting materials then their belief that they can actually perform the act of painting will inevitably diminish. Alternatively, if there is an abundance of opportunity, instruction, materials, and space to engage in creative work, then one's beliefs about what is possible will be directly impacted.

The opportunities for improving creative self-efficacy are not only dependent on the goals set by individuals, but also on the physical environment around those goals. Many creative activities and endeavors require at least basic materials to achieve meaningful engagement and the production of creative products. An enriching and resource-rich environment can set the stage for more robust mastery experiences, which will have a direct impact on one's sense of creative self-efficacy (Ganga et al., 2024). This relationship may be reciprocal though as many individuals who can demonstrate high confidence and potential are often rewarded with scholarships, sponsorships, and other material resources which then serve to further boost the sense of creative self-efficacy.

The people within one's physical environment are also important factors when considering creative self-efficacy. Apart from explicit encouragement or discouragement, role modeling and other behavioral cues can impact how we feel about ourselves. Fortunately, (Karwowski 2015) has examined the effects of creative peers in a physical space like a classroom and found no negative effects. In fact, they found that creative peers in a classroom can actually strengthen an individual's creative identity.

### Improving awareness of creative self-efficacy

Fortunately, self-efficacy is not a static trait. Self-efficacy judgments are elements of a dynamic system of self-beliefs. A wide variety of interventions, conducted in diverse psychosocial settings, have been shown to affect people's beliefs in their efficacy to handle the challenges of everyday life (Bandura, 2023; Lent et al., 1994). Numerous interventions have shown that targeted mastery experiences improve creative self-efficacy across educational and clinical settings (Liu et al., 2023; Zimmerman, 2000). The interventions that succeed implement the opposite of the downward spiral described above. Participants are given the opportunity and encouragement to try tasks which, upon completion, provide a sense of mastery (Artino, 2012).

Effective intentions for developing self-efficacy usually involve one or more of four specific criteria (Liu et al., 2023). The first and most robust are these first hand experiences of mastery. When participants are given the chance to set goals and then directly achieve those goals they can then use that experience to reshape their self-beliefs to include accomplishment and capability. Educational interventions aimed at improving creative awareness could provide ample opportunities for engaging with diverse creative mediums and practices. This will provide new mastery opportunities and improve the amount of creative experiences necessary to build higher creative self-efficacy.

In addition to these direct mastery experiences, self-efficacy can also be developed through vicarious experiences (Bartsch et al., 2012). Humans are particularly adept at social learning in which we witness others performing a behavior as a method to learn the behavior ourselves. Witnessing someone else achieve personal goals can often demonstrate just how possible certain tasks are. This can in turn help us to re-evaluate our own self beliefs to include more possibility and confidence in achieving our own goals.

Outside of direct mastery experiences and vicarious mastery experiences, self efficacy has been shown to improve through the process of verbal persuasion (Hendricks, 2016). This is a process in which both logical and emotion laden language is used to help an individual reshape their own self beliefs. These are some of the core processes involved in fields such as coaching, talk therapy, and motivational speaking.

Finally, emotional arousal has been shown to aid in the development of more efficacious self beliefs (Bartley and Ingram, 2018). People may evaluate their emotional arousal and overall bodily state when judging their efficacy for future performance. Situations that provide excitement and high energy emotions, or alternatively, calm and centering emotions, when paired with creative experiences may help to improve one's sense of creative self-efficacy.

### How creative self-efficacy is measured

(Tierney and Farmer 2002) have developed a three-item Creative Self-Efficacy Instrument, measured on a scale ranging from 1 (strongly disagree) to 5 (strongly agree) with items such as “I have confidence in my ability to solve problems creatively.” As discussed previously, creative self-efficacy and creative self-concept are often conflated, but measuring both may provide a way to make sure relevant details are not missed when assessing programs or interventions. The Short Scale of Creative Self (SSCS) (Karwowski et al., 2018) was designed to measure trait-like Creative Self-efficacy and Creative Personal Identity by asking respondents to indicate the degree to which they include the construct as part of who they are on a 5-point Likert scale.

The SSCS is composed of 11 items with six items measuring creative self-efficacy. Specifically, creative self-efficacy is described by the following six statements on the SSCS: “*I know I can efficiently solve even complicated problems”, “I trust my creative abilities”, “Compared with my friends, I am distinguished by my imagination and ingenuity”, “I have proved many times that I can cope with difficult situations”, “I am sure I can deal with problems requiring creative thinking,”* and “*I am good at proposing original solutions to problems*.” This scale has demonstrated satisfactory item-level discriminating power, an appropriate range of item difficulty, good item fit and functioning, adequate reliability, and internal construct validity (Shaw et al., 2021).

Three of the subscales from the Experience of Creativity Questionnaire also fit well with an examination of Creative Self-Efficacy. The Power and Pleasure scale examines experiences of heightened internal power and control that are mixed with a feeling of profound pleasure. The Distinct Experience scale differentiates everyday experiences from creative explorations, including greater emotional intensity and a heightened confidence and awareness of technical and expressive skills, and finally, The Clarity and Preparation scale explores feelings of certainty and clarity that inform the direction and meaning of the creative work; this is supported by adequate preparation to engage in the creative process (Nelson and Rawlings, 2009).

## Flow proneness

Flow is an intrinsically rewarding, fully absorbing creative state characterized by deep immersion, clear goals, immediate feedback, and a balance between challenge and skill (Csikszentmihalyi, 1999). When these preconditions are met the experience of flow includes a merging action and awareness, concentration on the task being executed, a sense of control while simultaneously losing a sense of self-consciousness, a perception that time has been altered, and the autotelic aspect of doing the activity for purely intrinsic pleasure and value (Jackson, 2004).

Flow is specifically related to an enjoyable experience of the creative process, in fact, in the literature it is often referred to as “Optimal Experience” (Csikszentmihalyi and Csikszentmihalyi, 1992). Thus, it is fitting as a central factor in a systems model focused on the experience of creative flourishing. Unfortunately, flow may not occur every time anyone engages in creativity. The experience of flow is in contrast to one of two opposing experiences of creativity. The first is boredom, which is typically characterized by challenges that are too low when compared with skills, a lack of clear goals, and low intrinsic motivation. The second is anxiety which usually involves challenges that are far beyond an individual's skills, goals that are too difficult to attain, and feedback that is difficult to interpret or implement. Flow Proneness refers to how often flow experiences take place in relation to these non-flow experiences of boredom and anxiety.

Flow Proneness is affected by many factors, however, studies have found that age, gender, socioeconomic status, and educational attainment only account for minimal variations in adults' flow experiences (Isham and Jackson, 2023; Thomson and Jaque, 2023). Accordingly, the rewards of flow appear to be available, in principle, across society and to diverse demographic groups. These findings suggest that flow experiences are not reserved solely for certain specific demographic groups and thus can represent an accessible route toward creative flourishing across society if the correct conditions are in place.

### How flow proneness relates to the cultural environment

According to the Systems Model of Creativity (Csikszentmihalyi, 2014), a stable cultural domain that will preserve and transmit ideas and forms throughout generations is a crucial part of creativity as a whole. Ideas, techniques, tools, and approaches to any creative act are considered part of the cultural domain. Some cultural domains are much more rich and detailed than others, for example the domain of painting as a creative practice has a much richer history, more numerous cultural techniques, and a broader set of tools than, say, 3D printing. A more enriching cultural environment can set the stage for flow to happen more easily with clearer goals, more options for feedback, and many tools with which to gain skill.

The cultural domain of a particular creative medium does not of course sprout from nowhere. It is forged by individuals who are innovating and creating new cultural ideas, tools, and artifacts, i.e., cultural memes. The inclusion of these memes into a creative domain is mediated by a field of experts and other social institutions, but nonetheless individuals who are forging new pathways for flow experiences make a direct impact on the culture in which they are embedded. For example scratching vinyl records grew from individual DJs in the 80s looking for ways to deepen the experience of the creative medium. Through a long and complex process of selection by the field through institutions like music venues, record labels, and music consumers, record scratching has become an established domain with international performances, competitions, and cultural resources. As individuals create new memes and expand a cultural domain, the richer environment facilitates flow by providing familiar tools, goals, and skills for emerging creatives.

### Improving awareness of flow proneness

Improving awareness around the necessary preconditions for flow, namely setting clear goals, receiving immediate feedback, and balancing challenges with personal skill, is vital for improving flow proneness in individuals (Goddard et al., 2023). Higher awareness of the importance of setting the stage for flow experiences could give individuals the resources and insight necessary to create more situations in which they get into flow, rather than leaving things to chance or the whim of the environment.

When these flow pre-conditions are not perfectly met, there is still room for awareness building. Programs and interventions aimed at improving awareness of flow proneness could use experiences of boredom and anxiety as learning opportunities. The ability to course-correct away from boredom and anxiety to move instead toward a state of flow is one of the most crucial skills related to creative flourishing. Interventions that provide coaching and educational scaffolding around recognizing the opportunities to make these course corrections will likely be more effective at improving flow proneness.

For example when a participant is feeling overwhelmed with a particular activity, scaffolding the activity to allow for simpler goals or tools that require less skill could help them re-enter a state of flow. Similarly, if a participant recognizes boredom with a particular activity, scaffolding around setting more demanding challenges or using tools that require more skill could help them ease back into a state of flow. This kind of scaffolding and awareness of flow processes could help individuals improve their flow proneness of their own volition, especially when paired with a sense of creative agency and creative self-efficacy.

### How flow proneness is measured

The Swedish Flow Proneness Questionnaire (SFPQ) (Ullén et al., 2012) appears to be one of the most popular ways to measure Flow Proneness (Gyurkovics et al., 2016; Mosing et al., 2012). It is a self-report measure exploring an individual's proneness to experience flow. It consists of three subscales with 7 items each and assesses flow during work, during leisure activities, and during maintenance. By capturing flow experiences across work and leisure, the SFPQ can reveal flow patterns across various contexts, illuminating areas to target for creative growth.

There is also The Dispositional Flow Scale (DSF-2) which is an excellent one-time measure to assess tendencies to experience flow (Jackson and Eklund, 2002). It effectively identifies personality trait-like characteristics and reveals general dispositional tendencies for specific activities as well as life in general. To accomplish this examination of an “Autotelic Personality” the researchers included a measure of the Five-factor traits using the Revised NEO Personality Inventory and combined that with a modified version of Csikszentmihalyi and Larson's (1984) Flow Questionnaire.

(Elnes and Sigmundsson 2023) have recently introduced a new 13 item scale called The General Flow Proneness Scale. Using a 5 point likert scale, questions include “*I enjoy challenging tasks/activities that require a lot of focus,” “When I am focused on a task/activity, I quickly tend to forget my surroundings,”* and “*I usually experience a good flow when I do something (things are neither too easy nor too difficult for me).”* The scale does not maintain a static interpretation of flow proneness, but rather works as a tool that may help understand the complexity of the concept of flow and autotelic personality.

Finally, there are also two subscales from the Experience of Creativity Questionnaire which would prove helpful in measuring flow proneness. The Creative Absorption scale includes a strong awareness of the creative action and a receptiveness to discovery, and The Creative Anxiety scale explores feelings of being more vulnerable during the creative process (Nelson and Rawlings, 2009). Both of these scales seek to examine aspects of creative experience that are directly related to flow proneness.

## Interactions across the systems model of creative flourishing

From a systems perspective we can see that creative agency and flow proneness have a direct reciprocal relationship (Zubair and Kamal, 2015). As we've explored in the previous sections, flow proneness requires clear goals in order for the flow state to occur more frequently and with more depth. These goals required for flow proneness stem directly from one's sense of creative agency. Working toward those goals and receiving direct and immediate feedback can feed into one's sense of creative agency with regard to self-reactiveness and self-reflection. This reciprocal relationship between flow proneness and creative agency is an important part of the systemic nature of creative flourishing.

Creative agency also has an important relationship with creative self-efficacy. As the processes of self-reaction and self-reflection take place we develop a sense of creative self-concept. This self-concept includes our own global beliefs about what kind of creative tasks we can accomplish. Both the process of setting personally meaningful goals and the process of mastering those goals require a sense of confidence. Reciprocally, acting as an effective agent can build creative self-efficacy, and mastering tasks with self-efficacy can build a stronger sense of creative agency (Tierney and Farmer, 2011).

The Creative Behavior as Agentic Action model (Karwowski and Beghetto, 2019) posits that creative confidence plays a mediating role between creative potential and creative behavior. This confidence could be measured in the form of creative self-concept or creative self-efficacy. More specifically, the model posits that creative potential works through creative confidence to influence creative behavior. This further supports the idea that creative agency and creative self-efficacy are systemically linked.

Flow proneness and creative self-efficacy are similarly linked. In order to have meaningful mastery experiences that build a stronger sense of self-efficacy one needs to have at least a modicum of control over the situation and themselves (Csikszentmihalyi, 2014). If there is no sense of control then there is no reason to attribute any mastery to oneself. Similarly, in order to attain a state of flow there needs to be a sense of control. This is primarily to maintain a balance between challenge and skill as well as for the opportunity for action and awareness to merge.

A lack of perceived control inhibits the balance between challenge and skill essential for achieving flow. The importance of a sense of control is how our aspects of flow proneness and creative self-efficacy interact. Getting into flow more often implies more control over creative processes, and more control over creative processes is directly implicated in mastery experiences which lead to increased creative self-efficacy (Kleppang et al., 2023).

Creative agency and creative self-efficacy together bolster flow proneness, fostering confidence and control in creative processes. Similarly, frequent flow states provide mastery experiences that heighten creative self-efficacy, reinforcing a feedback loop that supports creative agency. These reciprocal interactions is exactly why a systems-informed approach is fitting for examining the broad topic of creative flourishing.

## Discussion

The Systems Model of Creative Flourishing takes a phenomenological approach to understanding the complex interactions of psychological aspects inherent in positive creative experiences. Creative Agency allows an individual to set their own agenda as it relates to their creative practices (Royalty et al., 2013). This allows their goals and behaviors to become personally meaningful and intrinsically motivating, even as they interact with their social environment. Creative self-efficacy provides the foundation for enacting those meaningful personal choices in a way that facilitates mastery, rendering the autonomous goals as realized behaviors within the physical environment (Karwowski and Kaufman, 2017). Flow Proneness turns these realized behaviors into enjoyable and inherently valuable experiences that are absorbing, culturally embedded, intrinsically motivating, and endlessly engaging (Isham and Jackson, 2023).

When the needs of each of these aspects are met an individual can state that they are driven to create, they feel they have the ability to be creative, and they enjoy creating. Thus, creative flourishing is the result of the harmonious alignment of one's creative desires, self-perception of skills, and experiences within their social, cultural, and physical context. In integrating findings from Positive Psychology, social cognitive theory, and creativity studies, this model invites further research into how creative flourishing unfolds across diverse populations and contexts. It proposes that flourishing is not a static trait or state, but an emergent property arising from a dynamic system of beliefs, affective states, attentional orientations, and environmental affordances.

Positioning the model of creative flourishing alongside established theories such as (Csikszentmihalyi 2014)'s Systems Model, Shields' (2017) Subjective Creative Wellbeing Suprasystem, and the (Beresford et al. 2024) highlights both continuity and divergence. While each of these frameworks foregrounds different layers of systemic influence, the present model carves a distinct niche by centering the moment-to-moment experience of flourishing during the creative process. This focus is particularly relevant in educational, therapeutic, and developmental settings, where lived experience, personal meaning, and intrinsic motivation often matter more than external validation or domain advancement.

A challenge for any experiential model of creativity is that it may risk stating what seems “obvious.” However, the subjective clarity of these experiences in hindsight can obscure their systemic, dynamic, and emergent nature. The goal of this model is to offer a structured language to capture those fleeting, intuitive experiences in a way that informs research and supports flourishing in practice.

### Limitations

Limitations of this model include the limitations of any systems model in that the map is not the territory. To make a model of this sort is to intentionally simplify an incredibly complex landscape, which, while helpful for planning and understanding, can inevitably oversimplify or leave out important nuances. In this case, the systems model of creative flourishing may have an over-emphasis on individual phenomenological experience at the expense of more detailed environmental factors that play a role in creative flourishing.

A second limitation involves the balance between conceptual synthesis and empirical grounding. While the model draws upon a broad range of research, it has not yet been operationalized in a way that allows for direct empirical testing. Its current form remains theoretical and heuristic—designed more for reflection, interpretation, and application than for immediate measurement. Its purpose is to generate new research questions and guide practice. Future validation could involve empirically testing the strength and direction of relationships among the three components across diverse populations and creative domains.

Finally, this version of the model is presented without tailoring to specific populations. Its generality is a strength, but also a limitation when it comes to designing targeted interventions for youth, marginalized creatives, or professionals working in high-performance environments.

### Future directions

Future research on this particular model would benefit from systematic data collection before and after interventions aimed at improving the experiences of creative individuals. This may include educational programs, coaching, or arts-based therapies. Empirical studies can explore how the three components of Creative Flourishing interact dynamically in various populations, and whether changes in one domain (e.g., self-efficacy) reliably influence others (e.g., flow proneness). Interventions that intentionally cultivate creative agency—especially in educational or clinical contexts—could offer insight into the model's practical utility.

Researchers may also build on the previously described scales and questionnaires to develop a measurement tool to specifically examine creative flourishing directly. Future work might develop tools tailored to the model's components, potentially combining existing scales (e.g., Creative Self-Efficacy Scale, Short Dispositional Flow Scale) with new items that better capture phenomenological nuances. Finally, there is a need to explore how systems of oppression, marginalization, and gatekeeping intersect with individuals' ability to flourish creatively. Integrating social justice frameworks into creative flourishing research will be essential for ensuring that the model remains inclusive and equitable in practice.

## Conclusion

Creative Flourishing is a deeply human experience—emergent, relational, and alive in the interplay between self, environment, and possibility. The systems model proposed here aims to illuminate the underlying structures that shape such experiences, while also inviting practical application across psychology, education, and the arts. In centering lived experience and systemic awareness, the Systems Model of Creative Flourishing offers a foundation for future research, applied intervention, and reflective practice rooted in both individual experience and empirical evidence.
